# Rapid Detection of *Enterocytozoon hepatopenaei* Infection in Shrimp With a Real-Time Isothermal Recombinase Polymerase Amplification Assay

**DOI:** 10.3389/fcimb.2021.631960

**Published:** 2021-02-25

**Authors:** Chao Ma, Shihui Fan, Yu Wang, Haitao Yang, Yi Qiao, Ge Jiang, Mingsheng Lyu, Jingquan Dong, Hui Shen, Song Gao

**Affiliations:** ^1^ Jiangsu Key Laboratory of Marine Biological Resources and Environment, Jiangsu Key Laboratory of Marine Pharmaceutical Compound Screening, Co-Innovation Center of Jiangsu Marine Bio-industry Technology, School of Pharmacy, Jiangsu Ocean University, Lianyungang, China; ^2^ Jiangsu Institute of Oceanology and Marine Fisheries, Nantong, China

**Keywords:** *Enterocytozoon hepatopenaei*, recombinase polymerase amplification, recombination-dependent replication, spore wall protein gene, molecular detection

## Abstract

*Enterocytozoon hepatopenaei* (EHP) infection has become a significant threat in shrimp farming industry in recent years, causing major economic losses in Asian countries. As there are a lack of effective therapeutics, prevention of the infection with rapid and reliable pathogen detection methods is fundamental. Molecular detection methods based on polymerase chain reaction (PCR) and loop-mediated isothermal amplification (LAMP) have been developed, but improvements on detection speed and convenience are still in demand. The isothermal recombinase polymerase amplification (RPA) assay derived from the recombination-dependent DNA replication (RDR) mechanism of bacteriophage T4 is promising, but the previously developed RPA assay for EHP detection read the signal by gel electrophoresis, which restricted this application to laboratory conditions and hampered the sensitivity. The present study combined fluorescence analysis with the RPA system and developed a real-time RPA assay for the detection of EHP. The detection procedure was completed in 3–7 min at 39°C and showed good specificity. The sensitivity of 13 gene copies per reaction was comparable to the current PCR- and LAMP-based methods, and was much improved than the RPA assay analyzed by gel electrophoresis. For real clinical samples, detection results of the real-time RPA assay were 100% consistent with the industrial standard nested PCR assay. Because of the rapid detection speed and the simple procedure, the real-time RPA assay developed in this study can be easily assembled as an efficient and reliable on-site detection tool to help control EHP infection in shrimp farms.

## Introduction

Microsporidia are intracellular parasites which can infect a wide range of crustaceans and fish (Ning et al., 2019). Among Microsporidia, *Enterocytozoon hepatopenaei* (EHP) is an emerging pathogen and has been classified into the group of *Enterocytozoonidae*, suborder *Apansporoblastina*, phylum *Microsporidia*, and kingdom Fungi (Tourtip et al., 2009). In shrimp aquaculture industry, EHP can infect the hepatopancreas of different shrimp species including *Penaeus japonicas*, *Penaeus monodon*, and *Penaeus vannamei* (Tang et al., 2016; Thitamadee et al., 2016; Chaijarasphong et al., 2020). When infected, it causes stunted growth, soft shells, lethargy and white feces symptoms (Behera et al., 2019). In Asian countries including India, Thailand and China, EHP caused 10%–20% reduced production of shrimp annually leading to significant economic losses (Thamizhvanan et al., 2019). In the year 2015 in Jiangsu, China, EHP infections had caused 300 million CNY of loss for the shrimp farming industry. EHP has been considered a huge threat to farms in many shrimp-farming countries (Shen et al., 2019).

There are no specific clinical signs in EHP-infected shrimps, making it difficult to monitor EHP infection and to control the spreading. The feces of EHP-infected shrimp contain a large number of spores, which can infect healthy shrimp through horizontal transmission (Karthikeyan and Sudhakaran, 2019). In addition, there are no proven therapeutic methods for EHP infection (Chaijarasphong et al., 2020). Thus, it is very important to develop a rapid and simple detection method for EHP infection to prevent disease outbreaks and economic losses. To date, a number of diagnostic methods for the detection of EHP have been reported. These include loop-mediated isothermal amplification (LAMP), polymerase chain reaction (PCR), and quantitative PCR (qPCR) targeting the small subunit ribosomal RNA gene (SSU rRNA), nested PCR targeting the spore wall protein gene (*swp*) or *β-tubulin* gene, *in situ* hybridization assay, and histopathology (Jaroenlak et al., 2016; Han et al., 2018; Sathish et al., 2018; Piamsomboon et al., 2019). However, these methods have drawbacks like long detection time, need for trained personnel, and equipment dependence. Although the LAMP method takes only 45 min for the reaction, it still requires an accurate temperature-controlled machine (Sathish et al., 2018). These methods are not suitable for use in remote areas.

The isothermal recombinase polymerase amplification (RPA) assay derived from the recombination-dependent DNA replication (RDR) mechanism of bacteriophage T4 is a potentially suitable method (Li et al., 2018; Lobato and O’Sullivan, 2018; Dong et al., 2020; Hu et al., 2020; Wang et al., 2020). The RPA system uses several enzymes from bacteriophage T4, including the strand-exchange protein UvsX, the mediator protein UvsY, and the single-strand binding protein gp32, to mimic the bacteriophage T4 RDR system *in vitro*. UvsX and UvsY anchor on a DNA single strand and search for homologous sequences on another double-stranded DNA. Once a homologous sequence is found, a recombination event occurs and one strand of the double-stranded DNA is displaced by the single strand. The displaced strand is stabilized by gp32. The 3’-end of the displacing strand is extended by the *Bsu* DNA polymerase (Piepenburg et al., 2006). By mimicking the T4 RDR mechanism, the RPA system solves the low-temperature strand opening problem and amplifies the target DNA fragment isothermally at 37–42°C. Researchers have tried to apply the RPA technology to the detection of EHP infection in shrimp and, indeed, simplified the procedure because a thermocycler was no longer required. However, the analysis of amplification products by gel electrophoresis did not free the assay from the laboratory for field use, and the sensitivity was also limited by gel imaging tools (Zhou et al., 2020).

**Figure 1:**
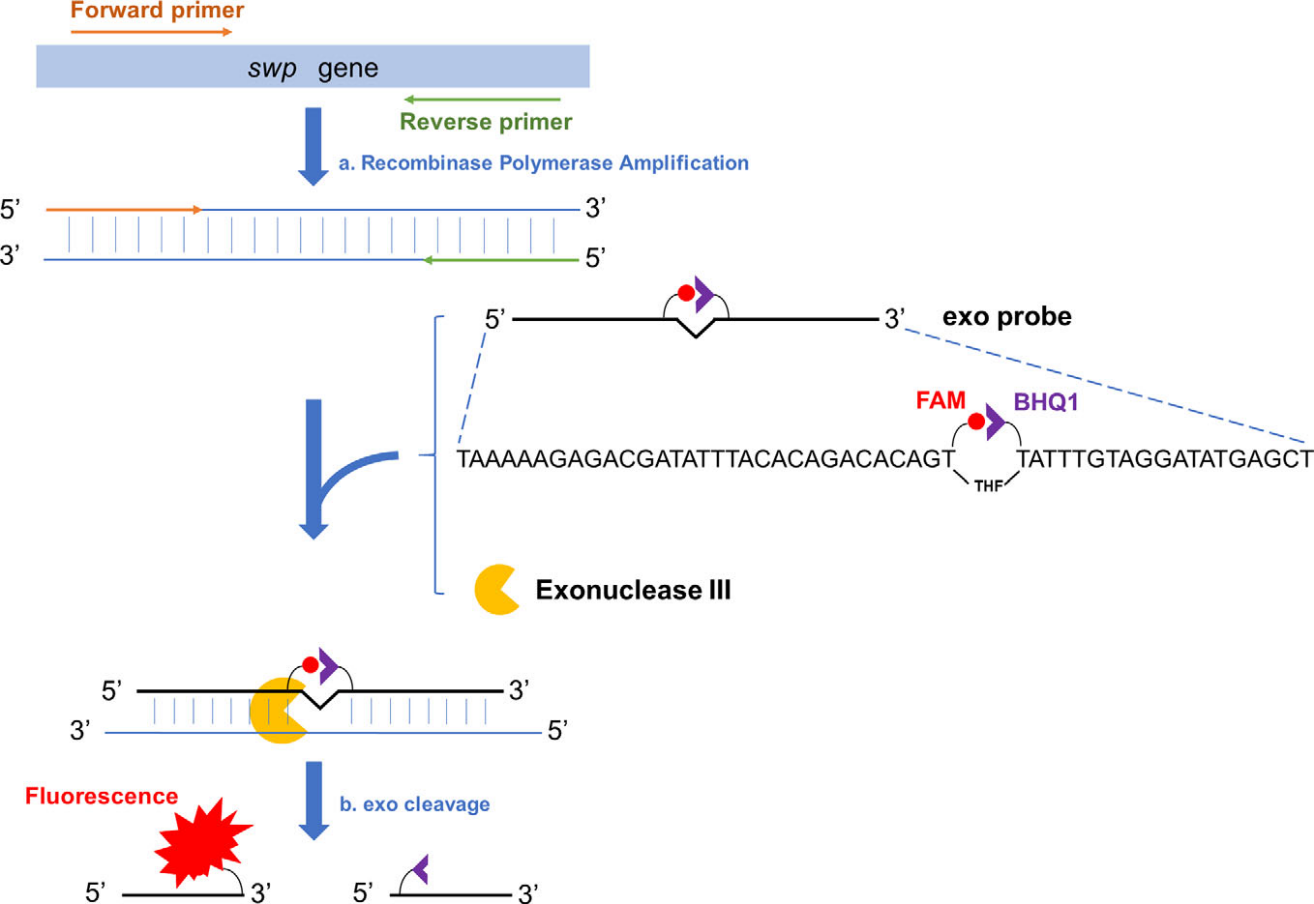
Schematic representation of the real-time RPA assay. Fragment of the target gene (*swp* gene in case of EHP detection) is isothermally amplified by recombinase polymerase amplification (event a). To one of the amplified strands, the exo probe anneals, and the Exonuclease III (exo) cleaves at the THF site once the bases flanking it have paired (event b). On the exo probe, the two T bases adjacent the THF site have been substituted by FAM-dT (fluorescent group) and BHQ1-dT (quenching group), respectively. The exo cleavage separates the two groups to allow fluorescence emission. There is a SpC3 group (not shown in this diagram) at the 3’-end of the exo probe to block the undesired extension of the probe.

The amplification products of RPA can be analyzed with better convenience by lateral flow chromatography or fluorescence (Li et al., 2018). Among these two options, fluorescence analysis makes real-time reading of the signal possible and this “real-time RPA” assay has a good combination of speed, portability, and accessibility. Briefly, the real time RPA reaction contains an “exo probe” that anneals to one of the amplified strands and recruits an exonuclease to cleave off the tetrahydrofuran (THF) substitution on the probe to separate the fluorophore and the quenching group at the two adjacent sites of THF on the probe, emitting the fluorescence signal (**Figure 1**) (Piepenburg et al., 2006).

In this study, a real-time RPA assay for rapid detection of EHP has been established. This method finished the detection in 10 min with good specificity. The limit of detection was 13 gene copies per reaction. Detection results were 100% consistent with the established nested PCR assay for real clinical samples. The real-time RPA assay is simple and reliable, and can be widely deployed for the detection of EHP infection in remote areas.

## Materials and Methods

### Infected Shrimp Samples, Bacterial Strains, and Clinical Samples

A collection of shrimps infected by *Enterocytozoon hepatopenaei* (EHP), white spot syndrome virus (WSSV), shrimp hemocyte iridescent virus (SHIV), and a *Vibrio parahaemolyticus* strain causing acute hepatopancreatic necrosis disease (AHPND) (referred to as *VP_AHPND_* in this study), and reference strains of *Vibrio vulnificus* and *Vibrio parahaemolyticus* (not AHPND-causative, referred to as non-*VP_AHPND_* in this study) were obtained from Jiangsu Institute of Oceanology and Marine Fisheries (Nantong, China). Clinical shrimp samples at different growing stages were collected from shrimp farms of different areas in China, including Qingdao, Rudong, Yancheng, Qidong, Lianyungang, and Rizhao. DNA of infected shrimp samples or clinical samples was extracted by the Magnetic Universal Genomic DNA Kit using the handheld 3^rd^ Gen. TGrinder (Tiangen Biotech Co., Ltd., Beijing, China) and quantified with a Qubit 4 fluorometer (Thermo Fisher Scientific Inc, Wilmington, DE, USA). DNA from the infected shrimp samples were confirmed for the presence of infection agents by qPCR as described previously (Zhu and Quan, 2012; Liu et al., 2018; Qiu et al., 2018; Zheng et al., 2018). The reference bacterial strains were confirmed by 16S rRNA sequencing (Hiergeist et al., 2016).

### Design of Primers and Probes

An NCBI Primer-BLAST (https://www.ncbi.nlm.nih.gov/tools/primer-blast) search was conducted using the FASTA sequence of the *swp* gene of EHP (GenBank accession no. KX258197.1). For parameter settings, the product size was set as minimum at 100 bp and maximum at 200 bp. The organism was set as *Enterocytozoon hepatopenaei* (taxid: 646526). The primer size was set at a minimum of 30 bases and a maximum of 35 bases. The primer GC content was set at a minimum of 20% and a maximum of 70%. The maximal self-complementarity was set as any at 4 and 3’ at 1. The maximal pair complementarity was set as any at 4 and 3’ at 1. Other parameters were set as default. For the probe design, the sequence defined by the primer pair was input into the Primer Premier 5 software. The size of the probe was set at a minimum of 46 bases and a maximum of 51 bases. The melting temperature (*Tm*) was set at a minimum of 57°C and a maximum of 63°C. The GC content was set at a minimum of 20% and a maximum of 80%. The maximum hairpin score was set as 1. The maximum primer-dimer score was set as 1. The maximum poly-X was set as 3. Other parameters were set as default. The probe had a C3 spacer (SpC3) at the 3’-end that could block strand extension and a tetrahydrofuran (THF) group at the middle to facilitate exonuclease III (exo) cutting. The two T bases adjacent the THF site were substituted by FAM (6-corboxy-fluorescein)-dT and BHQ1 (Black Hole Quencher 1)-dT (**Figure 1**). Primers and probes were synthesized by General Biosystems Co., Ltd., Anhui, China. The sequences are shown in **Table 1**.

**Table 1:** Primers and probe used in this study.

Method	Description	Sequence (5’-3’)	Length (bp)	Amplicon Size (bp)	Amplification target on Gene	GenBank No. of Gene
Real-time RPA	RPA-F	ACAATTTCAAACACTGTAAACCTTAAAGCA	30	176	79..254	KX258197.1 (*swp*)
	RPA-P	TAAAAAGAGACGATATTTACACAGACACAG[FAM-dT][THF][BHQ1-dT]ATTTGTAGGATATGAGCT	46			
	RPA-R	TCATTCATTTTCCTTTTATCTTCTGATATG	30			
Nested PCR (Jaroenlak et al., 2016)	SWP_1F	TTGCAGAGTGTTGTTAAGGGTTT	23	514	1..514	
	SWP_1R	CACGATGTGTCTTTGCAATTTTC	23			
	SWP_2F	TTGGCGGCACAATTCTCAAACA	22	147	38..184	
	SWP_2R	GCTGTTTGTCTCCAACTGTATTTGA	25			

### Construction of the Plasmid Standard

DNA extracted from the EHP-infected shrimp was used as the template and a pair of primers (SWP_1F and SWP_1R) was used for PCR amplification to obtain the target fragment of the *swp* gene (**Table 1**) (Jaroenlak et al., 2016). The PCR product was cloned into a pMD18-T vector (Takara Biomedical Technology Co., Ltd., Beijing, China) and verified by sequencing. The recombinant plasmid was extracted from the correct clone and quantified with a Qubit 4 fluorometer (Thermo Fisher Scientific Inc, Wilmington, DE, USA). The plasmid copy number was calculated based on its size (3,206 bp). The standard plasmid was tenfold serially diluted and used as templates for qPCR with the specific primers SWP_1F and SWP_2R targeting the *swp* gene fragment. The correlation of the *Ct* value with the copy number of the *swp* gene fragment was calculated from the qPCR results.

### Real-Time RPA Procedure

Real-time RPA reactions were performed according to the manufacturer instructions of the TwistAmp DNA Amplification exo Kit (TwistDx Inc., Maidenhead, United Kingdom). The reaction mixture contained 29.5 μl of rehydration buffer, 2.1 μl of each primer (10 μM), 0.6 μl of probe (10 μM), 12.2 μl of distilled water, 1 μl of the template, and a dried enzyme pellet. The reaction was initiated by adding 2.5 μl of magnesium acetate (280 mM) to the mixture. The reaction was conducted on a Roche LightCycler 480 II qPCR machine at 39°C in the FAM channel with signal reads at 15-s intervals for 20 min.

### Nested PCR

The nested PCR detection of EHP was performed as previously reported (Jaroenlak et al., 2016). Primers SWP_1F and SWP_1R were used for the 1^st^ PCR step with an expected amplicon size of 514 bp. From the reaction mixture of the 1^st^ PCR step, 1 μl was directly used for the 2^nd^ PCR step with primers SWP_2F and SWP_2R (**Table 1**). The expected amplicon size of the 2^nd^ PCR step was 147 bp. The amplicons were analyzed on a 1.5% agarose gel.

## Results

### Determination of Copy Number of the *swp* Gene Fragment in Extracted DNA

**Figure 2:**
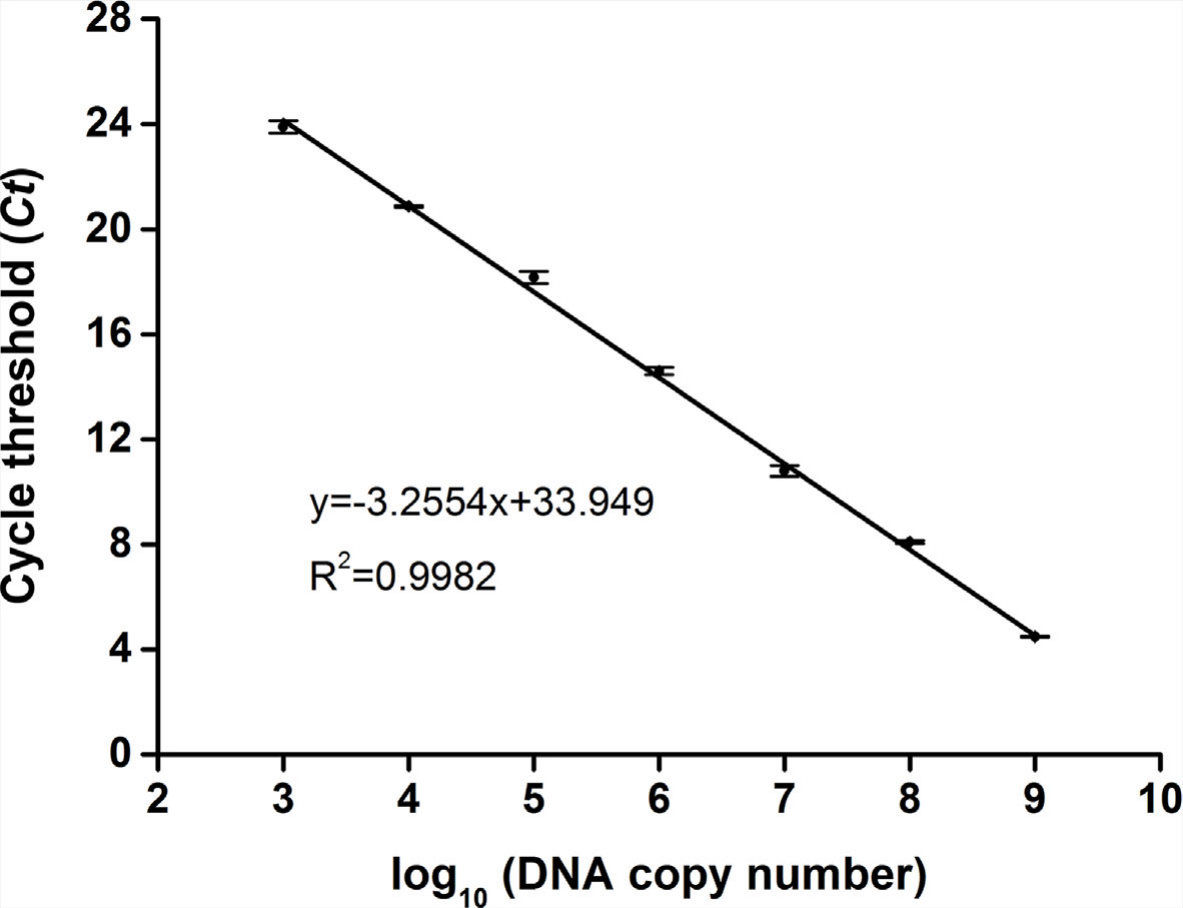
Standard curve of *swp* gene fragment by qPCR. For each reaction, 1 μl of the standard plasmid between 10^3^ and 10^9^ copies/μl was used as the template. The standard curve representing the correlation between the DNA copy number and the qPCR cycle threshold (*Ct*) value was built using GraphPad Prism 8.0 (GraphPad Software Inc, San Diego, CA). The function of the standard curve and the R^2^ value are indicated. The error bars represent the mean and standard error of three qPCR repeats.

To determine the copy number of the *swp* gene fragment in the extracted DNA of EHP-infected shrimp, a standard plasmid containing the gene fragment was constructed (**Supplementary Figure S1**), purified, and quantified spectrophotometrically. Tenfold serial dilutions of the standard plasmid from 10^9^ to 10^3^ copies/μl were used as the template for qPCR, and the *Ct* value for each concentration was determined. A standard curve was built showing a good correlation between the DNA copy number and the *Ct* value (R^2 =^ 0.9982) (**Figure 2**). This correlation was used to determine the copy number of the *swp* gene fragment in the extracted DNA of EHP-infected shrimp.

### Limit of Detection of the Real-Time RPA Assay

**Figure 3:**
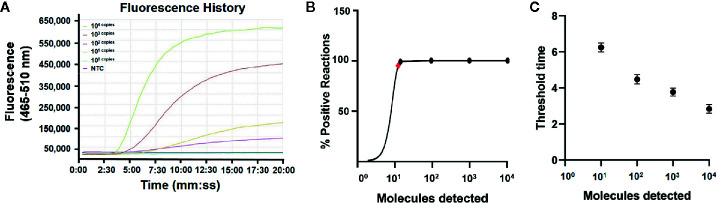
Limit of detection of the real-time RPA assay. **(A)** The fluorescence history diagram of the results of real-time RPA with different amounts (in copies) of the *swp* gene fragment. The amounts tested are indicated with different colors. The NTC is the no-template control. The diagram was one typical outcome of eight independent experiments. **(B)** Probit regression analysis of the data collected from the eight real-time RPA repeats using SPSS software (IBM, Armonk, NY, USA). The limit of detection at 95% probability (13 copies/reaction) is depicted by a red rhomboid. **(C)** Semi-logarithmic regression analysis of the data collected from the eight real-time RPA repeats using GraphPad Prism 8.0 (GraphPad Software Inc). The run time (threshold time) of the real-time RPA was 3–7 min for the templates at 10^4^–10^0^ copies/reaction.

DNA was extracted from the EHP-infected shrimp and quantified by qPCR using the standard curve. The quantified DNA was diluted to final concentrations of 10^4^ to 10^0^ copies/μl of the gene fragment and used to evaluate the limit of detection of the real-time RPA assay. The results showed that the signal of 10^1^ copies per reaction could be observed (**Figure 3A**). Reactions were conducted for eight independent repeats; 10^2^ copies and above per reaction were detected in all the eight repeats and 10^1^ copies per reaction were detected in seven of the eight repeats. A probit regression analysis was conducted and the limit of detection was calculated to be 13 copies/reaction in 95% of cases (**Figure 3B**). Semi-log regression analysis of the data of the eight repeats showed that the reaction time lengths of the real-time RPA assay to observe the signal were 3 to 7 min for 10^4^ to 10^1^ copies (**Figure 3C**).

### Detection Specificity of the Real-Time RPA Assay

To evaluate the specificity of the real-time RPA assay, shrimp samples infected with different viruses and microbes were tested (EHP, WSSV, SHIV, and *VP_AHPND_*). Also tested were reference strains of *V. vulnificus* and *V. parahaemolyticus* (*non-VP_AHPND_*). DNA extracted from the healthy shrimp (*P. vannamei*) was used as the control. The DNA concentrations were normalized to 10 ng/μl and used as the templates. For the two reference bacterial strains, incubation cultures of 10^6^ colony-forming unit (CFU)/ml were boiled at 100°C for 10 min and immediately used as the templates. Only the DNA from EHP-infected shrimp was positive, indicating good specificity of the real-time RPA assay toward EHP (**Table 2**).

**Table 2:** Information of shrimp samples and bacteria strains used in this study.

Infection agent	Sample type	Source/designation	Real-time RPA
*Enterocytozoon hepatopenaei* (EHP)	Infected shrimp (*Penaeus vannamei*)	Nantong, China	+
White spot syndrome virus (WSSV)	Infected shrimp (*Penaeus vannamei*)	Nantong, China	−
Shrimp hemocyte iridescent virus (SHIV)	Infected shrimp (*Penaeus vannamei*)	Nantong, China	−
*Vibrio parahaemolyticus* (*VP_AHPND_*)	Infected shrimp (*Penaeus vannamei*)	Nantong, China	−
*Vibrio vulnificus*	Reference strain	ATCC 27562	−
*Vibrio parahaemolyticus* (non-*VP_AHPND_*)	Reference strain	ATCC 17802	−
None	Healthy shrimp (*Penaeus vannamei*)	Nantong, China	−

(+, positive result; −, negative result).

### Application of the Real-Time RPA Assay for EHP Detection

A total of 32 clinical shrimp samples at different growth stages from several shrimp farms were tested for EHP with the real-time RPA assay and the nested PCR assay. A total of 22 EHP-positive samples were detected and the results of real-time RPA assay were 100% consistent with the nested PCR assay (**Tables 3**, **4**, and **Supplementary Figure S2**). These results indicated that the real-time RPA assay was effective for real clinical samples.

**Table 3:** Detection of *Enterocytozoon hepatopenaei* (EHP) infection in clinical samples.

No.	Sample type	Sample species	Length of shrimp (cm)	Source	Detection results	
					Nested PCR	Real-time RPA
1	Shrimp	*Penaeus vannamei*	0.5–3	Qingdao, China	+	+
2	Shrimp	*Penaeus vannamei*	0.5–3	Qingdao, China	+	+
3	Shrimp	*Penaeus vannamei*	0.5–3	Rudong, China	+	+
4	Shrimp	*Penaeus vannamei*	0.5–3	Rudong, China	+	+
5	Shrimp	*Penaeus vannamei*	4–5	Yancheng, China	+	+
6	Shrimp	*Penaeus vannamei*	8–9	Rudong, China	+	+
7	Shrimp	*Penaeus vannamei*	8–9	Rudong, China	+	+
8	Shrimp	*Penaeus vannamei*	0.5–3	Rudong, China	+	+
9	Shrimp	*Penaeus vannamei*	0.5–3	Rudong, China	−	−
10	Shrimp	*Penaeus vannamei*	5–6	Qidong, China	+	+
11	Shrimp	*Penaeus vannamei*	5–6	Yancheng, China	+	+
12	Shrimp	*Penaeus vannamei*	0.5–3	Lianyungang, China	+	+
13	Shrimp	*Penaeus vannamei*	0.5–3	Lianyungang, China	+	+
14	Shrimp	*Penaeus vannamei*	7–8	Yancheng, China	−	−
15	Shrimp	*Penaeus vannamei*	7–8	Yancheng, China	+	+
16	Shrimp	*Penaeus vannamei*	0.5–3	Rudong, China	+	+
17	Shrimp	*Penaeus vannamei*	0.5–3	Rudong, China	−	−
18	Shrimp	*Penaeus vannamei*	0.5–3	Rizhao, China	+	+
19	Shrimp	*Penaeus vannamei*	5–6	Rizhao, China	−	−
20	Shrimp	*Penaeus vannamei*	5–6	Lianyungang, China	+	+
21	Shrimp	*Penaeus vannamei*	0.5–3	Lianyungang, China	+	+
22	Shrimp	*Penaeus vannamei*	5–6	Rizhao, China	+	+
23	Shrimp	*Penaeus vannamei*	0.5–3	Rizhao, China	+	+
24	Shrimp	*Penaeus vannamei*	4–5	Qidong, China	−	−
25	Shrimp	*Penaeus vannamei*	7–8	Qidong, China	−	−
26	Shrimp	*Penaeus vannamei*	0.5–3	Rudong, China	+	+
27	Shrimp	*Penaeus vannamei*	0.5–3	Qidong, China	−	−
28	Shrimp	*Penaeus vannamei*	0.5–3	Rudong, China	+	+
29	Shrimp	*Penaeus vannamei*	0.5–3	Yancheng, China	−	−
30	Shrimp	*Penaeus vannamei*	4–5	Lianyungang, China	−	−
31	Shrimp	*Penaeus vannamei*	7–8	Rizhao, China	−	−
32	Shrimp	*Penaeus vannamei*	4–5	Rizhao, China	+	+

(+, positive result; −, negative result).

**Table 4:** Detection of *Enterocytozoon hepatopenaei* (EHP) infection in clinical samples (summarized).

Sample information	Number of samples	Number of positive samples detected	
		Nested PCR	Real-time RPA
Shrimp (0.5-3 cm)	17	13	13
Shrimp (4-5 cm)	4	2	2
Shrimp (5-6 cm)	5	4	4
Shrimp (7-8 cm)	4	1	1
Shrimp (8-9 cm)	2	2	2
Total	32	22	22

## Discussion

*Enterocytozoon hepatopenaei* (EHP) has emerged as a serious threat to shrimp aquaculture worldwide (Thitamadee et al., 2016). EHP infection in farmed shrimps does not cause mass mortality, but inflicts significant economic loss due to stunted growth and reduced feed consumption (Aranguren Caro et al., 2020) . Noting the lack of pharmacological interventions for EHP, a rapid and accurate detection assay of EHP infection in shrimp is significant for the shrimp farming industry as prevention is the only currently available control method. The currently available detection methods, including the nested PCR, qPCR, and LAMP, cannot fulfill the demand for rapidness and accessibility, especially in remote areas. Nested PCR and qPCR take several hours and require a precise temperature-controlled thermocycler. LAMP can finish the detection within an hour, but an accurately controlled thermal source is still needed.

The RDR mechanism of bacteriophage T4 provided a good strategy to open the double strands of DNA without the need for temperature elevation (Alberts and Frey, 1970; Kodadek et al., 1988). By assembly of the RDR system *in vitro* with UvsX, UvsY, and gp32 of bacteriophage T4 and a strand-displacing DNA polymerase from *Bacillus subtilis* (*Bsu*), the DNA amplification cycle, including strand opening, primer pairing, and chain extension, can be conducted under one constant temperature between 37 and 42°C. Exponential amplification of DNA is achieved very rapidly, usually within 30 min (Li et al., 2018). This RPA system derived from bacteriophage T4 has been commercialized and widely applied to molecular diagnosis for many infectious diseases (Dong et al., 2020; Hu et al., 2020; Wang et al., 2020). An effort has been made to apply the RPA technology for the detection of EHP infection that targeted the SSU rRNA gene, but the analysis of the amplification signal was performed by gel electrophoresis, which not only hampered the sensitivity (8 × 10^2^ gene copies per reaction) but also restrained the detection assay to the laboratory (Zhou et al., 2020).

Using fluorescence signal reading, this study described the development and evaluation of a real-time RPA assay for the detection of EHP infection in shrimp. The assay targeted the *swp* gene encoding the spore wall protein of EHP. This gene is considered a better molecular diagnosis biomarker than the SSU rRNA gene and has been used as the target in the previously established nested PCR method, which has been recognized as an industrial standard of shrimp farming (Jaroenlak et al., 2016). The results in this study confirmed the good specificity of this gene toward EHP detection.

The real-time RPA assay showed good detection sensitivity that was comparable to the nested PCR method and much better than the RPA assay using the gel electrophoresis analysis. The limit of detection was 13 copies/reaction in 95% of the cases. As referenced in other reports, the limit of detection of EHP was 10^1^ copies/reaction with the nested PCR method as well as with the qPCR- and LAMP-based methods (Liu et al., 2018; Ma et al., 2019). Comparing with the RPA assay using the gel electrophoresis analysis, the sensitivity of real-time RPA has improved for ~60 folds. Moreover, the real-time RPA results of real clinical samples are 100% consistent with the nested PCR method, indicating good reliability.

Besides the good sensitivity, the rapid and simple procedure is the advantage of the real-time RPA method. The detection procedure could be finished in 3–7 min at a conveniently low temperature of 39°C. For the pretreatment of the samples, DNA was extracted with a magnetic bead-based commercialized kit that only needs a magnet to perform the extraction. For the signal reading, although we used a qPCR machine to read the fluorescence signal in this study, portable tube scanners had been developed for field applications, such as the Genie III scanner from Beijing Suntrap Science & Technology Co., Ltd, China, the ESE Quant tube scanner from ESE Gmbh, Stockach, Germany, and the TwistDx tube-scanner from TwistDx Inc (Yi et al., 2014; Mondal et al., 2016; Geng et al., 2019). These small-sized, battery-powered fluorescence tube scanners are good replacements of qPCR machines for on-site real-time RPA detections. Because of the low dependence on equipment and power source, the real-time RPA assay can be easily assembled into a mobile suitcase laboratory for transport and use in the field (Mondal et al., 2016).

In conclusion, a real-time RPA assay was developed for rapid detection of EHP infection in shrimp. It can be applied as an efficient and reliable on-site detection tool to help control EHP infection in shrimp farms.

## Data Availability Statement

The original contributions presented in the study are included in the article/**Supplementary Material**. Further inquiries can be directed to the corresponding authors.

## Author Contributions

JD, HS, and SG designed the research. CM, SF, YW, and HY conducted the research. CM, YQ, GJ, and ML analyzed the data. CM and SG wrote the manuscript. All authors contributed to the article and approved the submitted version.

## Funding

This work was supported by grants from the National Natural Science Foundation of China (31470275), the Key Natural Science Research Project of the Jiangsu Higher Education Institutions of China (20KJA416002), the Fishery Science and Technology Innovation Program of China (Y2018-14), the Nantong Municipal Science and Technology Plan of China (GJZ17077), the Lianyungang Science and Technology Project of China (SF2003), the Science and Technology Project of Lianyungang High-tech Zone of China (HZ201901), the Open-end Funds of Jiangsu Key Laboratory of Marine Pharmaceutical Compound Screening (HY202004 and HY201805), the Postgraduate Research and Practice Innovation Program of Jiangsu Province (KYCX19_2296), and the Priority Academic Program Development of Jiangsu Higher Education Institutions of China.

## Conflict of Interest

The authors declare that the research was conducted in the absence of any commercial or financial relationships that could be construed as a potential conflict of interest.
