# Supplementary material for: Rapid Detection of Enterocytozoon hepatopenaei Infection in Shrimp With a Real-Time Isothermal Recombinase Polymerase Amplification Assay
Source: Front Cell Infect Microbiol. 2021 Feb 25;11:631960. doi: 10.3389/fcimb.2021.631960 (PMC7947341; doi:10.3389/fcimb.2021.631960)
Supplement: Supplementary file 1 [file DataSheet_1.pdf]

Supplementary File

Rapid detection of *Enterocytozoon hepatopenaei* infection in shrimp with a real-time isothermal recombinase polymerase amplification assay

Chao Ma[1,†], Shihui Fan[1,†], Yu Wang[1,†], Haitao Yang[1], Yi Qiao[2], Ge Jiang[2], Mingsheng Lyu[1], Jingquan Dong[1,*], Hui Shen[2,*], Song Gao[1,*]

[1]Jiangsu Key Laboratory of Marine Biological Resources and Environment, Jiangsu Key Laboratory of Marine Pharmaceutical Compound Screening, Co-Innovation Center of Jiangsu Marine Bio-industry Technology, School of Pharmacy, Jiangsu Ocean University, Lianyungang 222005, China

[2]Jiangsu Institute of Oceanology and Marine Fisheries, Nantong 226007, China

[†]These authors contributed equally to this paper.

*Corresponding authors.

Emails: 2018000029@jou.edu.cn (J. Dong), darkhui@163.com (H. Shen) and gaos@jou.edu.cn (S. Gao)

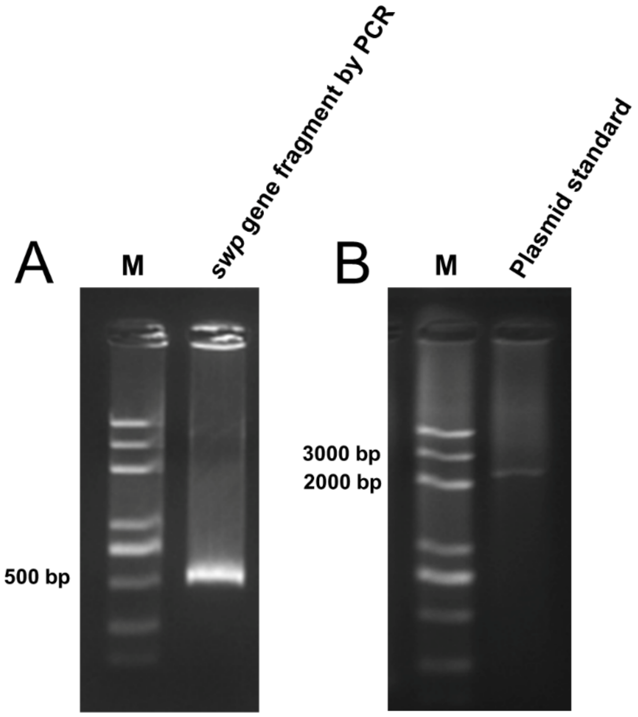

Figure S1 Construction of the plasmid standard. (A) Agarose gel image showing the PCR amplification product of the the *swp* gene fragment. (B) Agarose gel image showing the constructed plasmid standard. The M lanes are the DNA ladders. The positions of 500 bp, 2000 bp and 3000 bp bands of the DNA ladders are indicated.

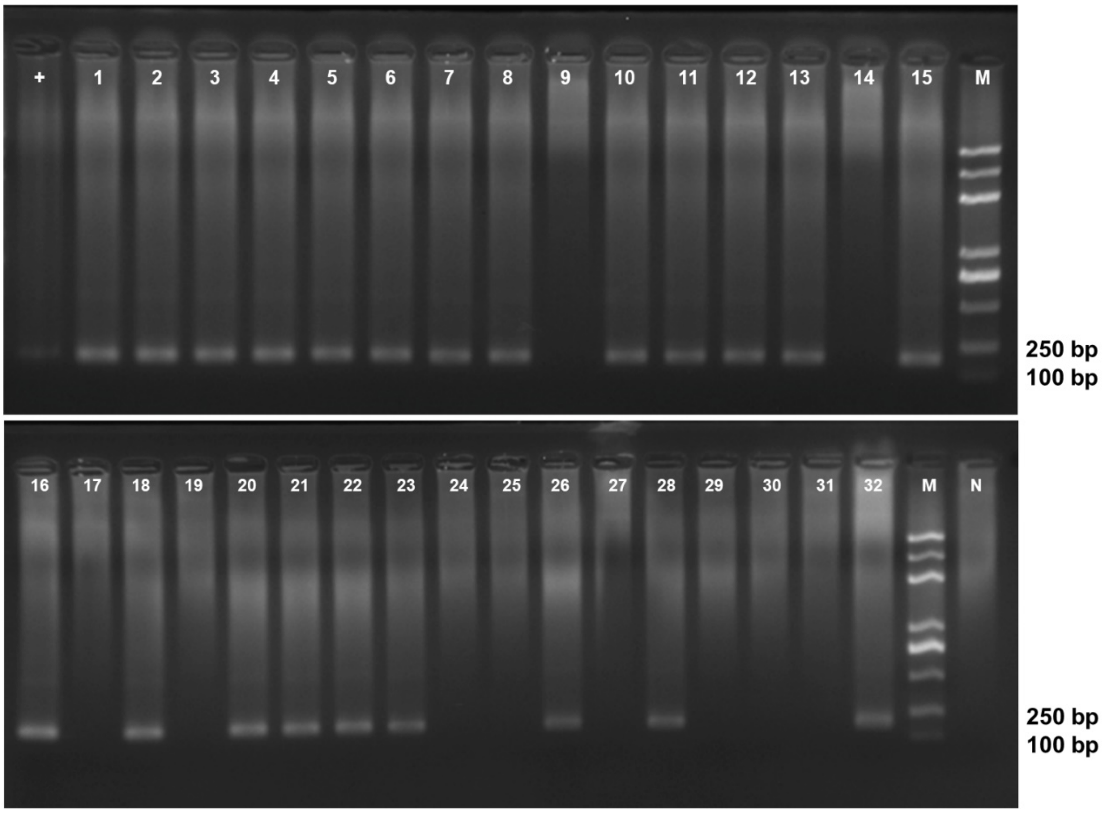

Figure S2 Detection of EHP in clinical samples by Nested PCR. Agarose gel images showing the detection results of nested PCR for the 32 clinical samples. The number of each clinical sample was indicated at the top of each lane. The plus (+) lane is the positive control (the plasmid standard was used as the template). The N lane is the no-template control. The M lanes are the DNA ladders. The positions of 100 bp and 250 bp bands of the DNA ladders are indicated.
